# Clinical Evaluation of ODIS-1 Orthodontic Operation and Image Quality of Digital Imaging System

**DOI:** 10.2174/0115734056345020250223150845

**Published:** 2025-03-11

**Authors:** Yuanli Zhang, Hong Huang, Chongzhi Yin, Guizhi Zhang, Yang Wang, Rui Gao, Jinlin Song

**Affiliations:** 1 College of Medical Technology, Chongqing Three Gorges Medical College, Chongqing 404120, China; 2 Chongqing Key Laboratory of Development and Utilization of Genuine Medicinal Materials in Three Gorges Reservoir Area, Chongqing 404120, China; 3 Stomatological Hospital of Chongqing Medical University, Chongqing 401147, China; 4 Chongqing Key Laboratory for Oral Diseases and Biomedical Sciences, Chongqing 401147, China

**Keywords:** Oral Digital System, Image Processing Technology, X-ray Imaging, Image Intensifier, K-nearest Neighbor Median Filtering

## Abstract

**Background:**

With the rapid development of computer technology, the application of digital technology to the display and processing of medical images has become a common concern. In recent years, oral digital imaging technology has received more and more attention.

**Objective:**

This paper mainly aims at the ODIS-1 oral digital imaging system to analyze and study the image quality and image aims at the ODIS-1 oral digital imaging system to analyze and study the image quality and processing technology, of which X-ray imaging is indispensable.

**Methods:**

In this paper, the ODIS-1 digital scanning technology is used to detect different types of dental tissues, and its application in diagnosing oral diseases is evaluated. This paper takes 320 inpatients as the research object and uses Kodak dental film to compare the image quality of different positions.

**Results:**

It is found that there is no significant difference in image quality between the maxillary anterior teeth and mandibular anterior teeth and the maxillary posterior teeth and mandibular posterior teeth (P>0.05); the image quality of maxillary anterior teeth, mandibular anterior teeth, and maxillary posterior teeth and mandibular teeth are significantly different (P<0.05); among the various positions of the ODIS-1 oral digital imaging system, the image quality of the anterior teeth area is the best, while the image quality of the maxillary posterior teeth area is the worst.

**Conclusion:**

However, the system has a variety of image post-processing functions, which can adjust the brightness and contrast of the image arbitrarily, select the area of interest in the image according to the detection requirements, and perform local amplification, edge enhancement, and other technologies to make the image achieve the best effect. In the case of poor image quality, the clarity of the image can be further improved through image post-processing and analysis.

## 1 INTRODUCTION

Digital imaging technology has the advantages of high sensitivity, fast imaging speed, real-time network transmission, fewer X-rays, and less radiation. Due to its large dynamic range, high density, rich layers, and the ability to perform post-processing, it has been widely used in China. X-ray digital image is a convenient and reliable non-destructive measurement method, widely used in many aspects. Digital imaging technology has a wide range of application values in the jaw, dental deformity, supernumerary teeth, impacted teeth, implant placement, traction, and so on. In this paper, the ODIS-1 digital image technology is used to evaluate and analyze the imaging quality of the system.

Digital dental technology is increasingly becoming an integral part of modern orthodontic practice, and research into oral digital imaging systems has become critical. Li G's study aimed to assess whether there are differences in the diagnostic accuracy of proximal caries between Chinese and American dentists. Dental education background does not affect the accuracy of digital radiographs in diagnosing proximal caries [1]. Koga-Ito C Y reviewed the efficacy of CAP and PAL in infectious diseases using *in vitro*, *ex vivo*, *in vivo*, and direct application methods. Significant progress has been made in wound healing, cancer treatment, and dental care [2]. Cai designed a new integrated model to compare the accuracy of two intraoral scanners (CEREC and TRIOS) and one extraoral scanner (SHINING). The results showed that the ensemble model can be scanned and imaged successfully. TRIOS outperforms CEREC and SHINING in accuracy, and this ensemble model can be used as a reference for intraoral scanner inspections [3]. The objective of Ozdede M was to evaluate the psychometric properties of the Turkish version of the revised questionnaire and to apply this questionnaire to Turkish dentists. The Turkish version of the Oral Radiology Control Questionnaire showed adequate psychometric properties. This suggested that it may be a valid and reliable tool for infection control assessment in Turkish dentists' oral radiology [4]. Mathew P. proposed a new classification system based on Computed Tomography (CT) evaluation, which enables surgeons to identify the extent of the defect and help execute the correct treatment plan [5]. These studies would be perfect if more consideration was given to the feelings of users.

With the development of image processing and network technology, more and more scholars have researched it. Sallam M proposed the promising application of ChatGPT in university education constructed specific and concise ChatGPT prompts based on expert group discussions and review of existing literature, and generated a response on February 25, 2023. The results indicate that the benefits of ChatGPT in medical education include improved personalized learning, clinical reasoning, and the possibility of understanding complex medical concepts [6]. Moghadam E T aimed to explore the types and extent of application of herbal products in oral health maintenance, including different areas of oral health such as dental caries, periodontal maintenance, microbial infections, oral cancer, and inflammation [7]. Bu Y proposed two artistic style classification schemes based on Scale-invariant Feature Transform (SIFT) features: the topic model and the pyramid matching model. Different from the discriminative model, to accomplish the classification task, the topic model used the topic distribution of images obtained from the topic learning process [8]. Akbar F H aimed to investigate and understand the relationship between dental tourism service quality, cultural equality satisfaction, and loyalty. He adopted a systematic review approach, guided by the Preferred Reporting Item for Systematic Reviews and Meta-Analyses (PRISMA) as the evaluation method. It was found that service quality and cultural equality have a related impact on dental tourism satisfaction and loyalty [9]. Roongruangsilp P reviewed and summarized the current literature on the concept, history, and initial applications of artificial intelligence in medicine and dentistry. Despite making many advances, artificial intelligence still has some limitations, but its opportunities are limitless as there is still enormous potential for continuous research in the fields of medicine and dentistry [10]. However, the processing process of these algorithms is relatively complicated, and the complex influencing factors of reality are not well considered.

The system studied in this paper can selectively display images, choosing mainly teeth or bones to highlight the part that the doctor wants to examine. Because humans have a stronger ability to identify subtle differences in simple backgrounds than those in complex backgrounds, they can more accurately determine the lesion location, which is not available in traditional apical radiography. The innovation of this paper is to combine the ODIS-1 system and image processing technology to realize a new type of oral digital imaging.

## 2 X-RAY IMAGE PROCESSING METHOD

### 2.1 Oral Digital Imaging System

Film imaging is a traditional dental imaging system. With the development of computer technology, a set of digital imaging systems has been produced. The ODIS-1 oral digital imaging system is divided into four parts, and their composition and function are shown in Fig. (**1**).

The X-ray detector converts the X-ray photons into electronic signals. After digital processing, the signals are sent to the computer, and the computer displays the picture directly on the screen. The system can work with the X-ray machine to generate oral X-ray images and store the images in the computer’s hard disk. The system can replace the existing X-ray photography technology, and accurately and quickly display the oral situation on the computer screen. At the same time, the stored data can be printed out, thereby shortening the waiting time of patients [11, 12].

The advantages of oral digital imaging systems are shown in Fig. (**2**).

Its digital image processing board and computer perform digital processing on the received signal, and the obtained image can be displayed on the computer screen in real time. The image data can be transmitted to the computer through the local area network, which is convenient for doctors to read. It eliminates the need for darkroom rinsing and imaging, reduces environmental pollution, reduces darkrooms, saves manpower and material resources, and greatly improves work efficiency.

Although there is no significant difference between the Class I image rate oral digital imaging system and film imaging technology, in the case of poor image quality, the system can adjust the clarity and brightness of the image, and reduce and enlarge part of the image to meet the requirements of Class I images. On the other hand, film imaging systems require repeated imaging, which can cause problems for patients who seek medical treatment. The system has functions such as image enlargement, reduction, length measurement, and data storage, which are convenient for clinicians to observe and analyze [13, 14]. The complexity of the background is reduced, and the influence of multiple layers of tissue overlapping on the imaging quality is avoided, which can effectively improve the identification of small lesions.

Currently, available data show that the radiation dose of digital imaging systems is lower than that of film imaging systems, and some even exceed 50%. Although the radiographic imaging system is unlikely to cause radiation damage when taking pictures, it is very valuable, especially for imaging workers, to minimize the radiation dose from a humane point of view.

However, its disadvantage is that it is expensive and requires multiple computer terminals. Although the area of the X-ray detector is only half of that of the X-ray dental film, the X-ray detector often brings discomfort to the patient due to its high hardness and is not easy to bend. Especially in the lower posterior teeth area, it cannot be placed, thus affecting the accuracy of the image. Because the patient is unwell and unable to coordinate, the image cannot be formed. In addition, when the patient wears the teeth, although the stored data can be printed out by the printer, the printed image is not as clear as the computer screen [15].

### 2.2 X-ray Source

The structure of the X-ray tube is shown in Fig. (**3**). There are three conditions for X-ray generation: the first is the light source that generates electrons - the cathode; the second is the way to accelerate the electrons - the tube voltage; the third is that the anode target is made of high-temperature tungsten wire, which is composed of a high-vacuum glass tube or a ceramic cover. After the cathode is energized, the filament is heated to release electrons, and the electric field at the head of the cathode gathers the electrons to generate a strong electric field and then hits the anode target at a very high frequency. The energy is exchanged with the anode target, part of which is converted into heat energy and part of which is converted into photons, thereby generating a series of X-rays [16]. The X-ray energy of an X-ray tube varies with the voltage in the tube and the current in the tube, and as the X-ray increases, the energy and penetration of the X-ray also increase. The regulation of the voltage in the tube is realized by adjusting the heating current of the filament, the current in the tube, and the primary voltage of the main transformer of the radiation device.

The relationship between X-ray photon energy and wavelength is shown in Formula (1):

**Table:** 

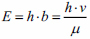	(1)

In Formula (1), *E* is the energy of each photon, and Planck's constant is *h* = 6.262 ×10^-34^ *J · D; b and µ* are the frequency and wavelength of X-ray photon emission; *v* = 3.0 × 10^8^ *m*/*s* representing the speed of light.

When light passes through an object, a series of interactions occur with atoms and electrons, namely Compton scattering, Rayleigh scattering, and electron effects. Absorption refers to the total conversion of the photon's energy into another energy, such as the kinetic energy of the electron, and the decay of the electron to its light. The so-called scattering means that when light passes through a target, its energy and properties would not change, but the direction of its propagation would be changed, resulting in Compton scattering and Rayleigh scattering [17, 18].

Non-destructive testing of X-rays is achieved through the absorption effect of photons. The initial energy *O*_0_ is a narrow and long mono-energy X-ray (a narrow beam is an unscattered beam, which is a beam composed of photons of the same energy), and the attenuation law of the narrow beam mono-energy X-ray is as follows:

**Table:** 

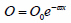	(2)

The formula is called Lambert-Beer. In Formula (2), x is the damping coefficient (that is, the material's ability to absorb light) when light passes through various materials. Generally speaking, the size of the light is inversely proportional to the intensity of the light and is proportional to the atomic number and density of the object. The denser the object, the harder it is to penetrate [19].

If the inspected material is defective, the dimension in the transmission direction is ∆*x*; the attenuation coefficient is *ϖ*′; the radiation intensity of the intact part is *O_α_*; the radiation intensity of the defective part is *O_α_*; the scattering rate is m; the overall intensity of the transmitted light is O, resulting in Formulas (3) - (5).

**Table:** 

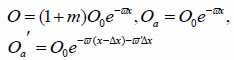	(3)

**Table:** 

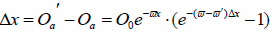	(4)

**Table:** 

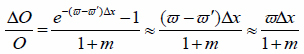	(5)

According to Formula (5), the light intensity difference between the transmitted light intensity and the unqualified point is the source of contrast. ∆*O*/*O* is the main contrast, which is related to factors such as transmission thickness, line attenuation coefficient, and scattering rate. This is also the basis for radiographic defect detection.

The image enhancement device adopts photoelectric conversion technology and is an important device in the ray digital image detection system. The system consists of three parts: input photoelectric conversion part, acceleration channel part, and output electro-optical conversion part. The structure is shown in Fig. (**4**).

Generally, materials that can scatter light have very little attenuation and have good absorption, such as aluminum and titanium, are used. By applying a flash lamp, a single crystal needle-shaped structure of sodium-containing cesium iodide (CsI: Na) is obtained on an aluminum base of about 0.5mm by vacuum evaporation. The scintillator is illuminated by X-rays and emits a green light that is transferred to the photocathode along the direction of crystal growth. The photocathode produces different numbers of photoelectrons under different visible fluorescence intensities.

The vacuum tube is also made of aluminum or titanium material to ensure that the photoelectrons can pass through the photocathode smoothly. The accelerating voltage in the tube is 25~35 KV. Due to the action of the focusing electrode, the high-speed photoelectron flow enters the surface of the scintillator through the focusing electrode, thereby making the electronic image smaller and improving the brightness. Usually, arc scintillators and photocathodes are used to reduce image distortion [20, 21].

After being hit by the accelerated electrons, the emitted scintillators produce visible green light. Generally, materials such as glass and optical fibers are used, and attention should be paid to anti-scattering and anti-reflection when designing. The CCD camera collects the output picture and observes and processes it. The specific workflow of the image intensifier can be simplified as Fig. (**5**):

The input screen of the image amplifier is divided into a single field of view, a dual field of view, and a three field of view. When the light intensity is under certain conditions, if a screen with a small field of view is used, the brightness of the image would decrease. However, the resolution would also be improved. Therefore, in the actual detection, an appropriate screen size should be selected.

The Charge-coupled Device (CCD) camera is a key part of the whole system, and its function is to convert the intensity of the visible light image incident on the photosensitive surface of the sensor into a regular and continuous output video signal. CCD cameras have the characteristics of small size, lightweight, high sensitivity, wide spectral response range, long life, low power consumption, shock resistance, shock resistance, etc., and have been widely used in aerospace, remote sensing, public security, transportation, electronics, robot vision, industrial production, and other fields [22].

After the X-ray image is converted into a visible light image by an image intensifier, it is accepted by a CCD camera and becomes a general image signal. Due to the small size of the output screen of the image amplifier, the visible light image it displays is only a few centimeters, so it must be enlarged. Due to the strong penetrability of X-rays, the X-rays that are not absorbed by the intensifier would be directly irradiated on the CCD, which would not only bring noise but also reduce the service life of the CCD. Therefore, a mirror is set in front of the CCD to reduce the effect of scattering on the CCD.

### 2.3 System Inhomogeneity Correction Scheme

Aiming at the non-uniformity, this paper uses the intensifier imaging technique to make a systematic correction.

The CCD undercurrent noise is mainly composed of background and peak. The background noise is Poisson distribution, which can be eliminated by the multi-frame stacking method. At different integration moments, the baffle lens acquires multiple images of the dark current spike noise and obtains the peak noise at each moment, and it is averaged through multiple samples to obtain the hourly peak noise *c*(*o*,*k*). If the image acquired by the CCD at time *t* under illumination is *U*(*o*,*k*), then the dark current peak noise is corrected by Formula (6), thereby obtaining the corrected image *U’*(*o*,*k*).

**Table:** 

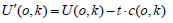	(6)

The inconsistency of the response of CCD pixels is mainly reflected in the inconsistency and invariance of the response ratio of each pixel, which is closely related to the production process of CCD. When the light response of the collected CCD is inconsistent, the camera lens is removed, and M pixels are collected under uniform light conditions to obtain *C*(*o*,*k*). The dark current is corrected by Formula (6) and *C’*(*o*,*k*) is obtained, and the light response inconsistency correction matrix expressed by Formula (7) is obtained.

**Table:** 

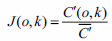	(7)

The image obtained by the CCD at the integration time *t* is *U*(*o*,*k*), and the image corrected for the dark current peak noise is *U’*(*o*,*k*). The photosensitive incompatibility correction matrix is corrected with Formula (7), and the resulting image is *U”*(*o*,*k*), represented by Formula (8).

**Table:** 

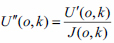	(8)

The inconsistent response of the image intensifier conversion screen is due to the inconsistency between the scintillation crystal particles on the screen and the X-ray conversion efficiency, which leads to gray fluctuations in the image collected by the CCD. This is a disadvantage of the screen itself. When the relative positions of the image intensifier, optics, and CCD camera are fixed, this non-uniformity is determined and can be corrected. The vignetting effect produced by the light source and the lens is related to the relative position of the light source, the intensifier conversion screen, and the intensity of the light source itself. After the hardware and relative positions of the detection system are determined, the above three factors can be comprehensively corrected when the measured element is not set.

The radiation source is adjusted to the actual required brightness. The detection system acquires M images and superimposes them to obtain an image *U* (*o*,*k*) *U* (*o*,*k*)s. Is corrected for the inconsistency between the dark current peak noise and the photosensitive response to obtain the corrected images *U”*(*o*,*k*) = (*U*(*o*,*k*) – *t*·*c*(*o*,*k*)) ·1/*j*(*o*,*k*). Among them, the number of *U*”(*o*,*k*) pixels is set to *m* × *n*, and the average value of all pixels *U”*(*o*,*k*) is obtained, as shown in the following Formula (9):

**Table:** 

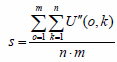	(9)

The three-factor combined correction matrix of inconsistent screen response, vignetting effect of ray source and lens is Formula (10):

**Table:** 

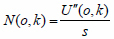	(10)

### 2.4 Image Smoothing

In the X-ray real-time imaging system, due to the unstable light source, scattering, etc., the dark current background noises in the CCD camera, errors in A/D conversion, emission, and human factors cause the clarity of the image to be unclear, and random noise in the image, which seriously affects the detection and identification of defects. The random noise in this system is mainly eliminated by frame stacking averaging and median filtering.

#### 2.4.1 Multi-frame Stacking Averaging Method

Multi-frame iterative averaging is a classic and effective method to eliminate random noise in the system. It can eliminate random noise under a certain number of overlapping frames under the premise of ensuring the signal-to-noise ratio of the image and ensuring that the edges of the image are not disturbed.

The basic idea of the multi-frame stacking noise reduction method is to stack multiple frames of multiple images in multiple images and obtain the average value. For ease of processing, the noise in the image is assumed to be additive noise. *g*(*o*,*k*) is an ideal image. *m*(*o*,*k*) is a random noise signal that is uncorrelated and has a mean of 0. *h*(*o*,*k*) is a noisy image superimposed with *m*(*o*,*k*) *h*(*o*,*k*). This can be expressed as:

**Table:** 

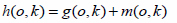	(11)

The object to be inspected is stationary, and the M frames of images are continuously accumulated and averaged. The result is shown in the following formulas:

**Table:** 

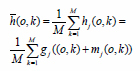	(12)

**Table:** 

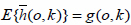	(13)

Through Formulas (12) and (13), it is obtained that after the overlapping operation of multiple frames, no useful image information is lost. However, the noise signal is reduced to 1/M, which indicates that the noise is effectively suppressed. Fig. (**6**) shows the grayscale values of the pixels in the 100th row scanned.

Compared with a single-frame image, the effect of the multi-frame superimposed image is smoother, and the random noise of a single frame can be effectively suppressed. It can be seen from the grayscale image in Fig. (**6**) that in a single image, due to the influence of random noise, there is a large roughness, and after iterative averaging, the effect is very good. The signal-to-noise ratio before and after the multi-frame average image is analyzed. The signal-to-noise ratio of each point (*o*,*k*) in a single frame image is obtained:

**Table:** 

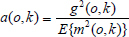	(14)

The signal-to-noise ratio after multi-frame stacking and averaging is:

**Table:** 

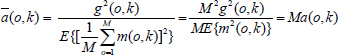	(15)

According to Formula (15), the multi-frame stacking and averaging operation improves the signal-to-noise ratio of the image by M times.

In theory, as the number of frames M increases, superimposed noise reduction can effectively reduce random noise. However, a large number of experimental results show that in the case of M>64, this noise reduction method not only has no obvious improvement but also has a certain impact on the details of the image. In practical applications, it is necessary to select an appropriate average number of superimposed frames according to the static and dynamic methods. If it is too small, the random noise in the image cannot be eliminated. M is usually 32 for static images, and dynamic detection causes errors in the image because the workpiece is moving. To solve this problem, when adjacent frames overlap, a deviation is set so that the defect areas between adjacent frames can be completely consistent.

#### 2.4.2 K-nearest Neighbor Median Filter

The K-nearest Neighbor (KNN) classification algorithm median filter algorithm is used in this paper. The concept of KNN median filtering is to replace the median value of K neighbors of neighboring pixels with neighboring pixels. When the pixel to be processed is a non-noise point, the method of approximating the gray value of adjacent points is to calculate according to the pixel value of the same area, which can not only maintain the smoothness of the image but also ensure the clarity of the image. If the pixel to be processed is a noise point, the suppression of the noise is achieved due to its isolation characteristics. The KNN median filtering method detects a noisy image as *g*(*o*,*k*):

**Table:** 

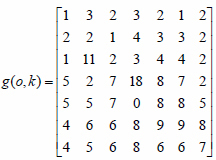	(16)

Two representative points are taken as examples. *g*(3,3) = 2 Is a noise-free point and *g*(5,4) = 0 is a noise point. Centered on *g*(3,3)=2, in the *M* × *M* template, L pixels that approximate the pixel value *g*(3,3) are selected, excluding *g*(3,3) itself. Usually, the pairing of L and M is fixed, and when M = 3, L = 3; when M = 5, L = 9; when M = 7, L = 25. A 5×5 template is constructed with g(3, 3) as the center, and nine points close to its pixel value are selected.

**Table:** 

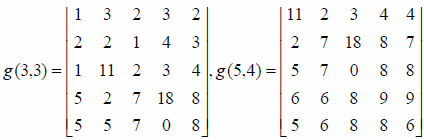	(17)

The 9 points that are close to the value of *g*(3,3) pixel are the 5 points in the 1st row, the first 3 points in the 2nd row, the 1st point in the 3rd row, and the 2nd point in the 4th row. After arranging, the median value is set to 2, and then *g*(3,3) = 2 there can be no noise.

*g*(5,4) = 0 is the center point, and the points adjacent to 9 pixels are *g*(1,2) = 2, *g*(1,3) = 3, *g*(1,4) = 4, *g*(1,5) = 4, *g*(2,1) = 2, *g*(3,1) = 5, *g*(4,1) = 6, *g*(4,2) = 6, *g*(5,1) = 5. If the median value is 4, then *g*(5,4) = 4 noise suppression.

Through 5 × 5 template filtering of the points in all the filtered areas of the entire image, the obtained image is as follows:

**Table:** 

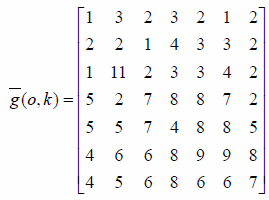	(18)

From the above operation results, it can be seen that the KNN median filter algorithm can effectively denoise the noise while ensuring the boundary of the image is preserved.

## 3 MATERIALS AND METHODS

### 3.1 Experiment And Evaluation Of Odis-1 Oral Digital Imaging System

According to the order of examination, 2022 patients in a hospital were selected, with a total of 320 patients. 160 were digitized dental film groups, and 160 were Kodak type. Each group was taken from the maxillary anterior teeth, mandibular anterior teeth, maxillary posterior teeth, and mandibular posterior teeth, with 40 dental films in each area. All teeth were done by a radiologist with intermediate technical qualifications. The basic conditions of the patients are shown in Table **1** and Fig. (**6**).

It can be seen from Table **1** that the gender ratio of males and females was close. In terms of age distribution, the patients were between 18 and 30 years old, accounting for 49.06%, followed by 31 to 50 years old, accounting for 26.25%.

In Fig. (**6a**), 47.50% of the patients checked their oral problems once a year, and only 3.75% of the patients never checked their oral problems before. The importance of oral problems needs to be strengthened. In Fig. (**6b**), 42.81% of patients had oral problems in recent months, and 37.50% of patients had oral problems for about a year. If left untreated, the problems may return or worsen.

The ODIS-1 digital image processing system is jointly developed by the Peking University School of Stomatology and the First Academy of the Ministry of Public Security. The size of the sensor is 37mm by 26.4mm and the number of pixels is 600x400. The X-ray sensor is placed at the position of the teeth corresponding to the patient's mouth, and the sensor automatically captures the X-ray signal and automatically triggers the camera function of the system. The digital image processing board and the computer digitally convert and process the received signal, and the obtained image can be displayed on the computer screen or printing machine in real time. The image is stored in the computer image database for users to refer to. The local area network is used to transmit the image data to the computer terminals of each clinic and department, which is convenient for clinicians to observe and analyze. The images are qualitatively and quantitatively analyzed using image processing technology, and remote consultation is carried out using the computer network.

The image quality criteria are as follows:

The clarity of the image is divided into three grades. Grade I: the image of the periodontal tissue at the subject site is clear; Grade II: the local image performance is not ideal, but meets the clinical requirements; Grade III: the image performance in most areas is not ideal and cannot meet the needs of clinicians.

Statistical method: A *X*^2^ pair of data was used for processing.

## 4 RESULTS

The ODIS-I digital imaging system requires less radiation dose than the film image. The X-ray image of the sensor in the patient's mouth is converted into a digital signal, which can be displayed on the computer screen. The software of the system realizes the functions of image magnification, enhancement, measurement, storage, etc. By measuring the image processing capabilities such as density, length, and area, the image can be analyzed qualitatively and quantitatively. However, compared with conventional X-ray films, the sensor of this system has strong rigidity and is not easy to bend, and during the imaging process, it is necessary to put a layer of sterile gloves on the outside of the sensor to avoid cross-infection. It is difficult to put the sensor in the proper place during the photographing process. Especially when photographing mandibular impacted teeth, it is even more difficult to get them in the correct position.

Table **2** shows the comparison of the image quality before digital dental image processing and Kodak dental image quality. After the *X*^2^_the_ test, there is a significant difference between the two (P<0.05).

The comparison of the image quality of the digital dental film image processing and the Kodak dental film image quality is shown in Table **3**. After the *X*^2^ test, the difference between the two was not statistically significant (P>0.05).

Fig. (**7**) shows the comparison of the image quality of the digital dental tablet for different positions. Fig. (**7a**) is the data for the relevant example, and Fig. (**7b**) is the ratio for each sample. The two groups were compared, and the results showed that there was no significant difference in the image quality between the maxillary anterior teeth and mandibular anterior teeth (P>0.05). The results showed that there were significant differences in image quality between the maxillary anterior teeth, mandibular anterior teeth, mandibular posterior teeth, and maxillary posterior teeth (P<0.05).

Fig. (**8**) shows a comparison of the image quality of different tooth positions taken by the Kodak dental film. There was no significant difference in image quality between the maxillary anterior teeth and the mandibular anterior teeth, and between the maxillary posterior teeth and the mandibular teeth after the *X*^2^ test (P>0.05). There were significant differences in image quality between maxillary anterior teeth, mandibular anterior teeth, maxillary posterior teeth, and mandibular posterior teeth (P<0.05). The quality of some images is similar, indicating that the imaging quality of the two systems is similar. Thus it can be concluded that post-image processing can compensate for the reduction in X-ray dose in the digital system, and the different quality of other images may be attributed to the detector position. Therefore, the conclusion is that the imaging quality of the digital system needs to be improved.

## 5 DISCUSSION

This study mainly conducted an in-depth analysis of the image quality and image processing technology of the ODIS-1 oral digital imaging system. The experimental results show that the image quality of the system varies in different tooth positions, among which the image quality of the anterior teeth is the best, and the image quality of the maxillary posterior teeth is relatively poor. This may be related to the specific structure of the teeth position, the projection angle of the X-ray, and the parameter setting of the imaging system. In future studies, these parameters can be further optimized to improve the overall image quality, especially the image clarity of the maxillary posterior teeth.

It is worth noting that although the image quality is insufficient in some tooth positions, the ODIS-1 system has rich image post-processing functions. These functions include adjusting the brightness and contrast of the image, selecting the area of interest for local magnification, edge enhancement, etc., which can effectively improve the image quality and improve the accuracy of diagnosis. Therefore, in practical applications, these post-processing techniques can be flexibly used to make up for the shortcomings of the original image quality.

In addition, this study also found that when using the ODIS-1 system for oral disease diagnosis, the patient's examination frequency and the duration of oral problems also have a certain impact on the diagnosis results. This suggests that in future research, in addition to focusing on the performance of the imaging system, the individual differences and examination habits of patients need to be considered to develop a more personalized and scientific examination plan.

In summary, the ODIS-1 oral digital imaging system has significant advantages and potential in clinical applications, but some issues need to be improved and optimized. By continuously optimizing imaging parameters, improving image processing technology, and considering individual differences in patients, it is hoped to further improve the diagnostic accuracy and clinical application value of the system and make greater contributions to the development of the field of oral medicine.

## CONCLUSION

Compared with the traditional apical radiograph, the disadvantage of the ODIS-1 digital oral imaging system is that the X-ray detector material used to collect digital teeth is hard, thick, and not easy to bend. In addition, to prevent cross-infection during filming, the probe must be put into the patient's mouth wearing sterile gloves to avoid making the patient feel uncomfortable and nauseous. Especially when there are teeth in the opposite dentition that are inclined towards the shallow floor of the mouth, narrow dental arch, and the palatal (lingual) side, or when the third molar is impacted horizontally, the pressure on the patient cannot be performed correctly. On the other hand, the traditional apical blade is soft, easy to bend, not easy to cause discomfort, and easy to locate and fix. The continuous improvement and improved image quality of the ODIS-1 oral digital imaging system can help clinicians play an important role in diagnosis, analysis, and scientific research.

## AUTHORS’ CONTRIBUTIONS

The authors confirm their contribution to the paper as follows: data collection: Y.Z., H.H., J.S.; analysis and interpretation of results: C.Y., G.Z., Y.W., R.G. All authors reviewed the results and approved the final version of the manuscript.

## Figures and Tables

**Fig. (1):**
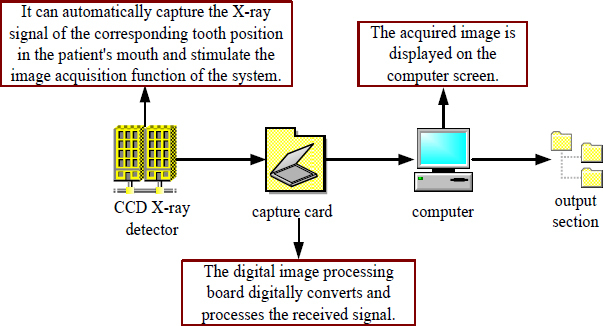
Composition of oral digital imaging system.

**Fig. (2):**
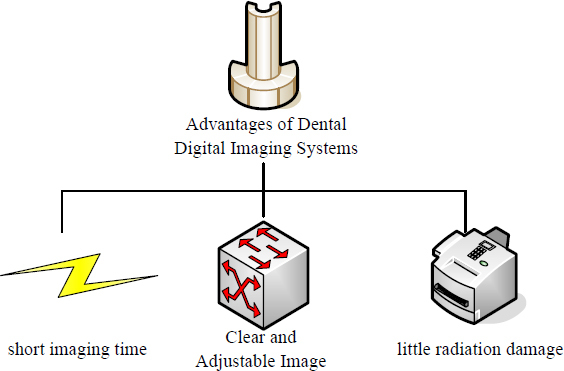
Advantages of oral digital imaging systems.

**Fig. (3):**
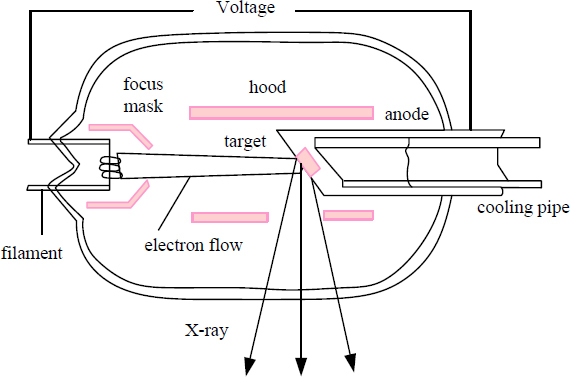
X-ray source structure diagram.

**Fig. (4):**
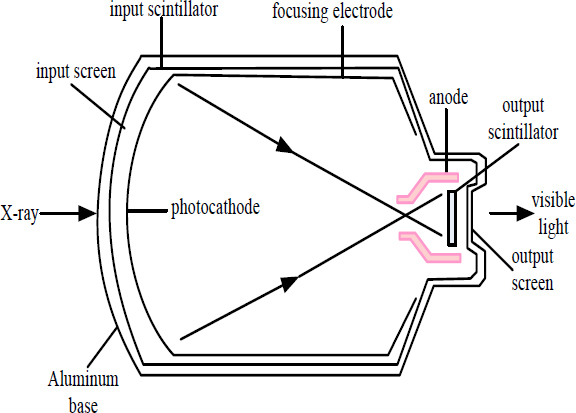
Schematic diagram of X-ray image enhancement.

**Fig. (5):**
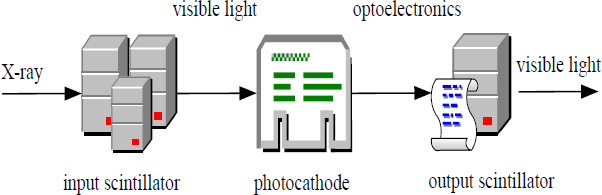
The working principle of the image intensifier.

**Fig. (6a,b):**
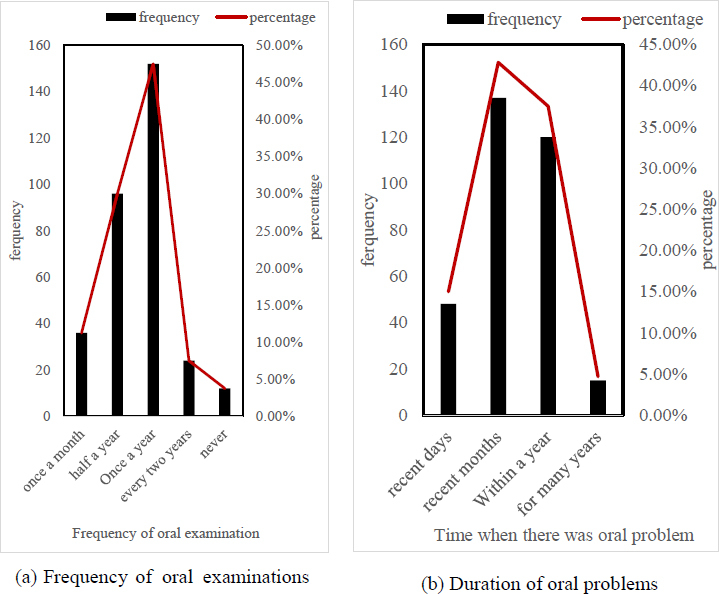
Partial oral information survey.

**Fig. (7a,b):**
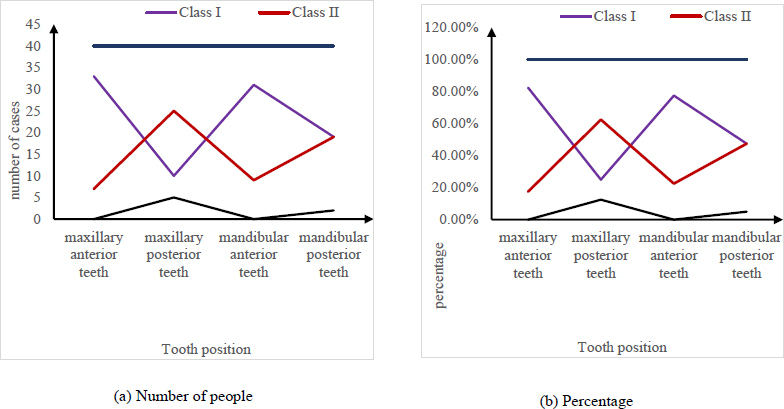
Comparison of image quality of different tooth positions captured by digital dental film.

**Fig. (8):**
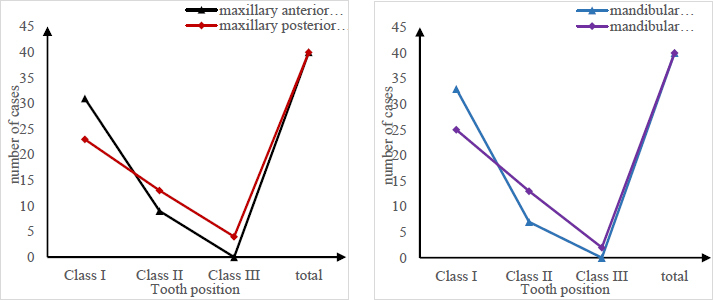
Comparison of image quality of different tooth positions taken with Kodak dental film.

**Table 1:** Demographic data.

**Demographics**		**Frequency**	**Percentage**
Gender	male	143	44.69%
	Female	177	55.31%
Age	under 18	32	10.00%
	18-30 years old	157	49.06%
	31-50 years old	84	26.25%
	51-60 years old	32	10.00%
	over 60 years old	15	4.69%

**Table 2:** Comparison of image quality before digital dental image processing and Kodak dental image quality.

**Group**	**Digital Dental Tablet**	**Kodak Dental Tablets**
Number of cases	160	160
Class I	124	140
Class II	34	20
Class III	2	0

**Table 3:** Comparison of image quality after digital dental image processing and Kodak dental image quality.

**Group**	**Digital Dental Tablet**	**Kodak Dental Tablets**
Number of cases	160	160
Class I	138	140
Class II	22	20
Class III	2	0

## Data Availability

The data and supportive information are available within the article.

## LIST OF ABBREVIATIONS

CCD: Charge-coupled Device
PRISMA: Preferred Reporting Item for Systematic Reviews and Meta-Analyses
SIFT: Scale-invariant Feature Transform
KNN: K-nearest Neighbor

## STANDARDS OF REPORTING

STROBE guidelines were followed.

## References

[1] Li G., Yang J. (2020). Diagnostic accuracy of approximal caries in digital radiograph by chinese and american dentists: An *in vivo* study.. Oral Surg. Oral Med. Oral Pathol. Oral Radiol..

[2] Koga-Ito C.Y., Kostov K.G., Miranda F.S., Milhan N.V.M., Azevedo Neto N.F., Nascimento F., Pessoa R.S. (2024). Cold atmospheric plasma as a therapeutic tool in medicine and dentistry.. Plasma Chem. Plasma Process..

[3] Cai H., Zhao B.C., Tian Y., Kim D.H., Sun Y., Lim H.K., Lee E.S., Jiang H.B. (2021). Design of a single-tooth model and its application in oral scan system assessment.. Scanning.

[4] Ozdede M., Akarslan Z., Altunkaynak B., Peker I. (2020). Turkish adaptation and implementation of the modified infection control questionnaire in intraoral digital imaging.. Eur. Oral Res..

[5] Mathew P., Kattimani V.S., Tiwari R.V.C., Iqbal M.S., Tabassum A., Syed K.G. (2020). New classification system for cleft alveolus: A computed tomography-based appraisal.. J. Contemp. Dent. Pract..

[6] Sallam M, Salim N A, Barakat M (2023). ChatGPT applications in medical, dental, pharmacy, and public health education: A descriptive study highlighting the advantages and limitations.. Narra J..

[7] Moghadam E.T., Yazdanian M., Tahmasebi E., Tebyanian H., Ranjbar R., Yazdanian A., Seifalian A., Tafazoli A. (2020). Current herbal medicine as an alternative treatment in dentistry: *In vitro*, *in vivo* and clinical studies.. Eur. J. Pharmacol..

[8] Bu Y. (2017). Research on the application of image processing technology based on SIFT features extraction in the retrieval and classification of artworks.. Rev. Fac. Ing..

[9] Akbar F H, Pasiga B D, Samad R (2020). The relationship between service quality, culture similarity to satisfaction and loyalty of medical (dental) tourism.. Sys. Rev. Pharm..

[10] Roongruangsilp P., Khongkhunthian P. (2022). Artificial intelligence with the application in medicine and dentistry.. J Osseointegr..

[11] Agrawal P., Nikhade P. (2022). Artificial intelligence in dentistry: Past, present, and future.. Cureus.

[12] Coulthard P. (2020). Dentistry and coronavirus (COVID-19) - Moral decision-making.. Br. Dent. J..

[13] Ossowska A., Kusiak A., Świetlik D. (2022). Artificial intelligence in dentistry—Narrative review.. Int. J. Environ. Res. Public Health.

[14] Meng L., Hua F., Bian Z. (2020). Coronavirus disease 2019 (COVID-19): Emerging and future challenges for dental and oral medicine.. J. Dent. Res..

[15] Sindhu D, Sindhu S . (2019). Image processing technology application for early detection and classification of plant diseases.. Int. J. Comput. Sci. Eng..

[16] Kumar A., Bhadauria H.S., Singh A. (2021). Descriptive analysis of dental X-ray images using various practical methods: A review.. PeerJ Comput. Sci..

[17] Muresan M.P., Barbara A.R., Nedevschi S. Teeth detection and dental problem classification in panoramic X-ray images using deep learning and image processing techniques.. 2020 IEEE 16th International Conference on Intelligent Computer Communication and Processing (ICCP).

[18] Karagyozov P., Boeva I., Tishkov I. (2019). Role of digital single-operator cholangioscopy in the diagnosis and treatment of biliary disorders.. World J. Gastrointest. Endosc..

[19] Johnson B., Laprade C., Broome A., Mol A., Platin E. (2020). Bitewing dosimetry of 3-dimensional intraoral tomosynthesis dental x-ray imaging system.. Oral Surg. Oral Med. Oral Pathol. Oral Radiol..

[20] Yoon S., Jung H.J., Knowles J.C., Lee H.H. (2021). Digital image correlation in dental materials and related research: A review.. Dent. Mater..

[21] Reddy C.H., Anitha M., Feroz A., Chandrashekar L., Sudarshan R., Vigneswary (2017). Digital imaging analysis with fractal dimension in oral leukoplakia.. Asian J Res Med Pharm Sci..

[22] Coelho-Silva F., Gaêta-Araujo H., Rosado L.P.L., Freitas D.Q., Haiter-Neto F., de-Azevedo-Vaz S.L. (2021). Distortion or magnification? An *in vitro* cone-beam CT study of dimensional changes of objects with different compositions.. Dentomaxillofac. Radiol..

